# Thyroid hormones associate with risk of incident chronic kidney disease and rapid decline in renal function: a prospective investigation

**DOI:** 10.1186/s12967-016-1081-8

**Published:** 2016-12-03

**Authors:** Xiaolin Huang, Lin Ding, Kui Peng, Lin Lin, Tiange Wang, Zhiyun Zhao, Yu Xu, Jieli Lu, Yuhong Chen, Weiqing Wang, Yufang Bi, Guang Ning, Min Xu

**Affiliations:** 1 State Key Laboratory of Medical Genomics, Key Laboratory for Endocrine and Metabolic Diseases of Ministry of Health, National Clinical Research Center for Metabolic Diseases, Collaborative Innovation Center of Systems Biomedicine and Shanghai Clinical Center for Endocrine and Metabolic Diseases, Rui-Jin Hospital, Shanghai Jiao-Tong University School of Medicine, Shanghai, 200025 China; 2 Department of Endocrine and Metabolic Diseases, Shanghai Institute of Endocrine and Metabolic Diseases, Rui-Jin Hospital, Shanghai Jiao-Tong University School of Medicine, 197 Rui-Jin 2nd Road, Shanghai, 200025 China

**Keywords:** Thyroid hormones, Chronic kidney disease, Renal function, Glomerular filtration rate

## Abstract

**Background:**

Thyroid hormones have been associated with renal dysfunction in cross-sectional studies. However, prospective studies exploring the effect of thyroid hormones on renal function decline were sparse and got contradictive results. We aimed to prospectively explore the associations of thyroid hormones with incident chronic kidney disease (CKD) and rapid decline in estimated glomerular filtration rate (eGFR) in Chinese adults.

**Methods:**

The participants were from a community-based cohort including 2103 individuals aged 40 years or above without CKD at baseline. Thyroid-stimulating hormone (TSH), free triiodothyronine (FT3) and free thyroxin (FT4) were measured by radioimmunoassay at baseline. Serum creatinine, urinary creatinine and albumin were measured at baseline and follow-up. CKD was defined as eGFR <60 ml/min/1.73 m^2^ or urinary albumin-to-creatinine ratio ≥30 mg/g. Rapid eGFR decline was defined as an annual eGFR decline >3 ml/min/1.73 m^2^.

**Results:**

During 4 years of follow-up, 198 participants developed CKD and 165 experienced rapid eGFR decline. Compared to tertile 1, tertile 3 of FT4 levels were associated with 1.88-folds (95% confidence interval [CI], 1.27–2.77) increased risk of incident CKD; and 1.64-folds (95% CI, 1.07–2.50) increased risk of rapid eGFR decline (both *P* for trend ≤0.02), after adjustment for confounders. Each 1-pmol/l of FT4 was associated with 12% increased risk of incident CKD and 10% of rapid eGFR decline. Among the incident CKD individuals, FT4 was significantly associated with higher risk of concurrent complications and further outcomes of CKD. We did not find associations of FT3 or TSH with CKD or rapid eGFR decline.

**Conclusions:**

Higher FT4, but not TSH and FT3, was associated with increased risk of incident CKD and rapid eGFR decline in middle-aged and elderly Chinese.

## Background

Chronic kidney disease (CKD) is manifested as the presence of albuminuria, impaired glomerular filtration rate, or both [1]. It has been found to be associated with an increased risk of cardiovascular morbidity and mortality, bringing heavy social and economic burden subsequently [2–4]. Now, CKD is affecting approximately 13.1% of Americans and 10.8% of Chinese adults [5, 6]. Although diabetes, hypertension, and family history of CKD, etc., have been established as risk factors for CKD [7, 8], disclosure of more novel risk factors for CKD or decline in estimated glomerular filtration rate (eGFR), which is a surrogate for development of CKD [9], may help extensively understand the pathogenesis of CKD and develop new prevention strategies for CKD.

Thyroid hormones, which are crucial for regulation of human body’s physiological actions, were found influencing GFR, renal blood flow, tubular secretory and re-absorptive processes as well [10–12]. Previous studies reported that overt and subclinical hypothyroidism were associated with higher levels of serum creatinine, reduced eGFR and increased risk of CKD [13–16]. Hyperthyroidism, however, can also lead to or accelerate the development of CKD [17]. In fact, most of the evidence was derived from cross-sectional studies [18, 19]. Prospective studies exploring the effect of thyroid hormones on renal function decline were sparse and got contradictive results [20, 21]. One study showed that normal-to-high levels of thyroid-stimulating hormone (TSH) and normal-to-low levels of free triiodothyronine (FT3) were associated with an increased risk of incident CKD in euthyroid individuals [20]. While the other reported non-significant association between thyroid dysfunction and changes in renal function among older adults [21].

In the present investigation, we aimed to explore prospectively the associations of thyroid hormones with risk of incident CKD and rapid eGFR decline; in addition, we examined the associations with predicted risks for concurrent complications and further outcomes of CKD, among the incident CKD participants.

## Methods

### Study population and design

All participants of the present study were recruited from Songnan Community, Baoshan district, Shanghai, in two stages as reported previously [22, 23]. In stage 1, 10,185 residents aged 40 years old or above were enrolled to the screening examination in June and July 2008. According to different levels of fasting plasma glucose (FPG), all participants were categorized into three groups: normal glucose regulation, impaired glucose regulation and diabetes. In stage 2, from June to August 2009, 4012 participants were randomly selected from the three groups on a ratio of 1.44 (normal glucose regulation) to 1.2 (impaired glucose regulation) to 1.0 (diabetes) because subjects with lower glucose levels were expected to have a lower participation rate than those with higher glucose levels, for a more comprehensive survey.

**Fig. 1:**
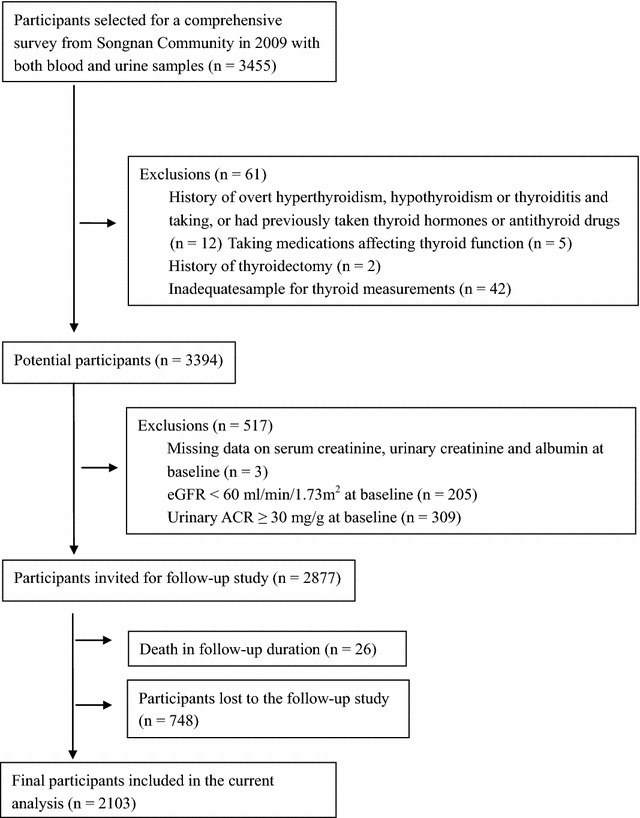
Flow chart of study participants. *eGFR* estimated glomerular filtration rate, *ACR* albumin–creatinine-ratio

Among the 4012 participants, 3455 participants had both blood and urine samples. For the present analysis, participants were excluded sequentially as follows, with a history of thyroid dysfunction (history of overt hyperthyroidism, hypothyroidism or thyroiditis, taking or had previously taken thyroxine or antithyroid drugs, n = 12), currently taking medications which affecting thyroid function (n = 5), with a history of thyroidectomy (n = 2), with inadequate sample for thyroid measurements (n = 42), with missing data on serum creatinine, urinary creatinine and albumin (n = 3), and with eGFR <60 ml/min/1.73 m^2^ (n = 205) or urinary albumin-to-creatinine ratio (ACR) ≥30 mg/g (n = 309). After exclusion, 2877 participants were eligible. From March to June 2013, all the 2877 participants were invited to have a follow-up study. 26 participants died during the follow-up. Eventually, 2103 participants responded and participated in the follow-up study (details on Fig. 1).

Written informed consent was obtained by each participant and the study protocol was approved by the Institutional Review Board of Rui-Jin Hospital.

### Data collection

A questionnaire survey and anthropometric measurements were conducted both at the baseline and follow-up visit. Sociodemographics, lifestyle factors and medical history were collected by trained physicians with a standard questionnaire via face-to-face interview. Current drinkers and smokers were defined as those who consumed alcohol at least once a week, and those who smoked a cigarette per day or seven cigarettes per week in the past 6 months, respectively. The information on body weight and height was measured with participants wearing light weight clothing and no shoes. Body mass index (BMI) was calculated as weight in kilograms divided into squared height in meters. Waist circumference was measured at umbilical level in a standing position. After at least 5 min rest, blood pressure was measured three times with 1-min interval using an automated electronic device (OMRON Model HEM-752 FUZZY, Omron Company, Dalian, China), at the non-dominant arm in a seated position. The average of the three readings was used for analysis.

### Biochemical measurements

After an at least 10 h overnight fast, the venous sample was collected and a 75-g oral glucose tolerance test (OGTT) was performed for each participant both at the baseline and follow-up visit. Levels of serum triglyceride, high-density lipoprotein cholesterol (HDL-c), low-density lipoprotein cholesterol (LDL-c), total cholesterol (TC), and serum creatinine (Cr) were measured by an autoanalyser (ADVIA-1650 Chemistry System, Bayer Corporation, Germany). FPG and OGTT 2-h plasma glucose were measured with using the glucose oxidase method on an autoanalyser (ADVIA-1650 Chemistry System, Bayer Corporation, Germany), and hemoglobin A1c (HbA1c) was determined by high-performance liquid chromatography (BIO-RAD D-10, USA). eGFR was evaluated using the 2009 chronic kidney disease epidemiology collaboration (CKD-EPI) equation [24] according to the recommendation in the 2012 kidney disease: improving global outcomes (KDIGO) clinical practice guideline for the evaluation and management of CKD [1]:

(1) Females: Cr ≤ 0.7 mg/dl, eGFR = 144 × (Cr/0.7)^−0.329^ × (0.993)^age^; Cr > 0.7 mg/dl, eGFR = 144 × (Cr/0.7)^−1.209^ × (0.993)^age^. (2) Males: Cr ≤ 0.9 mg/dl, eGFR = 141 × (Cr/0.9)^−0.411^ × (0.993)^age^; Cr > 0.9mg/dl, eGFR = 141 × (Cr/0.9)^−1.209^ × (0.993)^age^.

A first-voided, early-morning spot urine was collected to measure the urinary albumin and creatinine using rate nephelometry (Beckman Coulter, Fullerton, CA) and alkaline nitroxanthic acid method, respectively. Urinary ACR was calculated as milligrams of urinary albumin excretion per gram of urinary creatinine.

### Measurements of thyroid function

Levels of serum free thyroxin (FT4), FT3, TSH, thyroid peroxidase antibody (TPOAb) and thyroglobulin antibody (TGAb) were measured at the baseline by the Clinical Laboratory for Endocrinology, Shanghai Institution of Endocrine and Metabolic Disease, which was certified by College of American Pathologists, and assessed using chemiluminescent microparticle immunoassay method by Architect system (Abbott Laboratories, Abbott Park, IL). The reference ranges of FT4, FT3 and TSH were 9.01–19.04 pmol/l, 2.62–6.49 pmol/l and 0.35–4.94 μIU/ml, respectively, with the corresponding inter-assay coefficients of variations (CV) of 2.6–5.3%, 4.7–8.0%, and 3.1–3.4%. The reference range was <5.61 IU/ml for TPOAb with an inter-assay CV of 4.3–6.8% and <4.11 IU/ml for TGAb with an inter-assay CV of 3.2–5.2% [23, 25].

### Outcomes

In present study, the primary outcome was incident CKD which was defined as the presence of eGFR <60 ml/min/1.73 m^2^ or urinary ACR ≥30 mg/g at the follow-up visit [26]. The secondary outcome was rapid eGFR decline which was defined as an annual decline [(eGFR at follow up—eGFR at baseline)/follow-up period (years)] in eGFR >3 ml/min/1.73 m^2^ [27].

Furthermore, among the participants who developed CKD, we predicted the risks of concurrent complications and further outcomes of CKD by combining eGFR with albuminuria in the 2012 KDIGO clinical practice guideline for the evaluation and management of CKD [1]. It was classified into four colored risk zones for prognosis: low risk, green; moderately increased risk, yellow; high risk, orange; and very high risk, red.

### Statistics analysis

All statistical analyses were performed by using SAS version 9.3 (SAS Institute Inc, Cary, NC, USA). The values of serum TSH, TPOAb, TGAb, triglyceride, FPG and HbA1c, and urinary ACR were normalized by logarithmic transformation because of skewed distributions. Continuous variables were shown as means ± standard deviations (SDs) for those with normal distribution and were shown as medians (interquartile ranges) for those with skewed distribution. All categorical variables were presented as numbers (proportions). Thyroid function measurements were treated as continuous variables with one-unit increase or categorical variables by tertiles: <13.60, 13.60–14.83 and ≥14.84 pmol/l for serum FT4; <4.43, 4.43–4.83 and ≥4.84 pmol/l for serum FT3; <1.151, 1.151–1.860 and ≥1.861 μIU/ml for serum TSH.

Comparisons of baseline characteristics between non-CKD and incident CKD individuals were performed by using *t* test for continuous variables and Chi-square test for categorical variables. *P* values for trend for percentages of incident CKD and rapid eGFR decline were tested by using Cochran–Mantel–Haenszel (CMH) method among tertiles of serum FT4.

Multivariable logistic regression analysis was used to evaluate the associations of serum FT4, as well as serum FT3 and TSH, with the development of CKD and rapid eGFR decline. In model 1, age, sex and BMI at baseline were adjusted; in model 2, triglyceride, HDL-c, urinary ACR (for incident CKD only) and eGFR, current smoking and drinking, diabetes and hypertension status, and usage of antidiabetic or antihypertensive drugs at baseline were further adjusted based on model 1; in model 3, serum TPOAb and TGAb levels at baseline were further adjusted based on model 2. Additionally, we used the multinomial logit regression analysis to evaluate the associations of baseline serum FT4 levels with the predicted risks for future outcomes of CKD, among incident CKD individuals.

Significance tests were two-tailed and a *P* value less than 0.05 was considered to be statistically significant.

## Results

### Characteristics of the participants at baseline

A total of 2103 participants free of CKD at baseline were included in current analyses. The mean age was 59.3 years old and 39.3% were males. During the 4-year follow-up period, 198 (9.4%) participants developed CKD and 165 (7.8%) experienced rapid eGFR decline. Table 1 shows baseline characteristics of the participants according to incident CKD status. Participants who developed CKD were older, had higher BMI, waist circumference, blood pressure, serum triglyceride, FPG, HbA1c and urinary ACR, and lower level of eGFR (all *P* values <0.05). 26.3% of those who developed CKD at follow-up were taking antidiabetic drugs and 31.8% were using antihypertensive drugs, significantly higher than that of non-CKD participants (all *P* values <0.05). In particular, levels of serum FT4, but not FT3 and TSH, were higher among the incident CKD participants than non-CKD ones (*P* < 0.0001). No significant differences were observed in levels of HDL-c, LDL-c, TC, TPOAb, TGAb, sex distribution, or status of current smoking and drinking at baseline between incident CKD and non-CKD participants.

**Table 1:** Baseline characteristics of the participants according to incident CKD status

Characteristics	Non-CKD	Incident CKD	*P* value
n, %	1905 (90.6)	198 (9.4)	–
Age (years)	58.9 ± 8.9	63.1 ± 10.1	<0.0001
Male (n, %)	753 (39.5)	73 (36.9)	0.47
BMI (kg/m^2^)	24.7 ± 3.4	25.5 ± 3.8	0.0006
Waist circumference (cm)	86.3 ± 9.4	89.5 ± 9.7	<0.0001
Current drinker (n, %)	344 (18.1)	40 (20.2)	0.58
Current smoker (n, %)	382 (20.1)	39 (19.7)	0.81
SBP (mmHg)	134 ± 24	144 ± 27	<0.0001
DBP (mmHg)	78 ± 10	81 ± 10	0.001
Triglyceride (mmol/l)	1.40 (0.96–2.02)	1.48 (1.01–2.41)	0.02
LDL-C (mmol/l)	2.40 ± 0.68	2.35 ± 0.68	0.30
HDL-C (mmol/l)	1.37 ± 0.30	1.34 ± 0.31	0.31
Total cholesterol (mmol/l)	5.15 ± 0.96	5.11 ± 1.05	0.64
FPG (mmol/l)	5.1 (4.7–5.8)	5.6 (5.0–7.0)	<0.0001
HbA1c (%)	6.1 (5.7–6.5)	6.4 (5.9–7.6)	<0.0001
TSH (μIU/ml)	1.44 (1.03–2.14)	1.44 (1.01–2.07)	0.35
FT3 (pmol/l)	4.65 ± 0.55	4.72 ± 1.11	0.16
FT4 (pmol/l)	14.27 ± 1.80	14.85 ± 2.36	<0.0001
TPOAb (IU/ml)	0.30 (0.17–0.73)	0.32 (0.18–0.71)	0.55
TGAb (IU/ml)	1.06 (0.71–2.44)	0.93 (0.71–1.91)	0.09
eGFR (ml/min/1.73 m^2^)	92.4 ± 12.2	88.9 ± 14.4	0.0001
Urinary ACR (mg/g)	4.67 (2.39–8.71)	9.57 (4.41–16.87)	<0.0001
Use of antidiabetic drugs (n, %)	208 (10.9)	52 (26.3)	<0.0001
Use of antihypertensive drugs (n, %)	426 (22.4)	63 (31.8)	0.003

*P* values are calculated by *t* test for continuous variables and Chi-square test for categorical variables

Data are means ± standard deviations or medians (interquartile ranges) for continuous variables, and numbers (proportions) for categorical variables

*ACR* albumin-to-creatinine ratio, *BMI* body mass index, *DBP* diastolic blood pressure, *eGFR* estimated glomerular filtration rate, *FT3* free triiodothyronine, *FT4* free thyroxine, *FPG* fasting plasma glucose, *HDL-c* high-density lipoprotein cholesterol, *HbA1c* hemoglobin A1c, *LDL-c* low-density lipoprotein cholesterol, *SBP* systolic blood pressure, *TSH* thyroid-stimulating hormone, *TPOAb* thyroid peroxidase antibody, *TGAb* thyroglobulin antibody

### Association of thyroid hormones with incident CKD

The incidence of CKD gradually increased according to serum FT4 tertiles: 6.7, 8.6 and 12.9% for tertile 1, tertile 2 and tertile 3, respectively. As compared to tertile 1, tertile 3 of FT4 levels was associated with 88% increased risk of developing CKD (95% confidence interval [CI] 1.27–2.77; *P* for trend = 0.001), after adjustment for the confounding factors (Model 3). The continuous variable analysis showed similar results. Each 1-pmol/l of FT4 was associated with 12% (95% CI 1.05–1.20) increased risk of incident CKD. We did not found a significant association between serum FT3 or TSH and incident CKD (Table 2).

**Table 2:** Association of thyroid hormone levels with incident CKD

	Case/number (%)	Model 1	Model 2	Model 3
Serum FT4				
Tertile 1	47/699 (6.7)	1.00	1.00	1.00
Tertile 2	60/701 (8.6)	1.28 (0.86–1.91)	1.26 (0.83–1.91)	1.24 (0.82–1.88)
Tertile 3	91/703 (12.9)	1.97 (1.36–2.86)	1.91 (1.30–2.81)	1.88 (1.27–2.77)
*P* for trend	<0.0001	0.0003	0.0008	0.001
Each 1-pmol/l increase in FT4	198/2103 (9.4)	1.13 (1.05–1.21)	1.12 (1.04–1.20)	1.12 (1.05–1.20)
Serum FT3				
Tertile 1	65/687 (9.5)	1.00	1.00	1.00
Tertile 2	68/712 (9.6)	1.07 (0.74–1.53)	1.11 (0.76–1.62)	1.12 (0.76–1.63)
Tertile 3	65/704 (9.2)	1.09 (0.75–1.59)	1.06 (0.72–1.57)	1.04 (0.70–1.54)
*P* for trend	0.88	0.64	0.76	0.86
Each 1-pmol/l increase in FT3	198/2103 (9.4)	1.23 (1.01–1.49)	1.21 (0.99–1.49)	1.20 (0.98–1.47)
Serum TSH				
Tertile 1	64/700 (9.1)	1.00	1.00	1.00
Tertile 2	74/700 (10.6)	1.13 (0.79–1.61)	1.31 (0.90–1.90)	1.34 (0.92–1.94)
Tertile 3	60/703 (8.5)	0.86 (0.59–1.26)	0.99 (0.66–1.47)	1.04 (0.69–1.56)
*P* for trend	0.70	0.45	0.99	0.80
Each 1-μIU/ml increase in TSH	198/2103 (9.4)	0.99 (0.91–1.07)	0.99 (0.92–1.07)	1.00 (0.94–1.07)

Model 1: adjusted for age, sex, BMI at baseline

Model 2: further adjusted for triglyceride, HDL-c, diabetes and hypertension status, current smoking and drinking status, use of antihypertensive drugs, use of antidiabetic drugs, urinary ACR, eGFR at baseline

Model 3: further adjusted for TPOAb, TGAb at baseline

*P* for trend values for percentages of incident CKD are calculated by using Cochran–Mantel–Haenszel (CMH) method

Data are odds ratios (95% confidence intervals)

*ACR* albumin-to-creatinine ratio, *BMI* body mass index, *CKD* chronic kidney disease, *eGFR* estimated glomerular filtration rate, *FT3* free triiodothyronine, *FT4* free thyroxine, *HDL-c* high-density lipoprotein cholesterol, *TSH* thyroid-stimulating hormone, *TPOAb* thyroid peroxidase antibody, *TGAb* thyroglobulin antibody

### Association of thyroid hormones with rapid eGFR decline during follow-up

During follow-up, the eGFR levels of non-CKD individuals significantly increased, while those of incident CKD participants were decreased (all *P* values <0.05). In follow-up study, eGFR of non-CKD and incident CKD individuals were 93.0 ± 10.6 and 84.1 ± 18.1 ml/min/1.73 m^2^, respectively. With the increment of serum FT4 tertiles, the percentages of rapid eGFR decline gradually increased (*P* for trend = 0.0004). Multivariable logistic regressions showed that the odds ratio (OR) for rapid eGFR decline in the highest FT4 tertile versus the lowest tertile was 1.64 (95% CI 1.07–2.50), after adjustment for various confounders. Each 1-pmol/l increase of serum FT4 was associated with 10% increased risk of rapid eGFR decline. We did not found a significant association between serum FT3 or TSH and risk of rapid eGFR decline (Table 3).

**Table 3:** Association of thyroid hormones with rapid eGFR decline during follow-up

	Case/number (%)	Model 1	Model 2	Model 3
Serum FT4				
Tertile 1	40/699 (5.7)	1.00	1.00	1.00
Tertile 2	56/701 (8.0)	1.40 (0.92–2.14)	1.38 (0.89–2.12)	1.40 (0.91–2.16)
Tertile 3	69/703 (9.8)	1.72 (1.15–2.59)	1.61 (1.06–2.45)	1.64 (1.07–2.50)
*P* for trend	0.0004	0.009	0.03	0.02
Each 1-pmol/l increase in FT4	165/2103 (7.9)	1.12 (1.05–1.21)	1.10 (1.02–1.18)	1.10 (1.03–1.18)
Serum FT3				
Tertile 1	55/687 (8.0)	1.00	1.00	1.00
Tertile 2	69/712 (9.7)	1.29 (0.89–1.87)	1.35 (0.91–1.99)	1.36 (0.92–2.00)
Tertile 3	41/704 (5.8)	0.79 (0.51–1.21)	0.84 (0.54–1.30)	0.83 (0.53–1.30)
*P* for trend	0.025	0.33	0.50	0.49
Each 1-pmol/l increase in FT3	165/2103 (7.9)	1.17 (0.95–1.44)	1.14 (0.93–1.40)	1.13 (0.92–1.39)
Serum TSH				
Tertile 1	63/700 (9.0)	1.00	1.00	1.00
Tertile 2	53/700 (7.6)	0.77 (0.53–1.14)	0.85 (0.57–1.26)	0.85 (0.57–1.26)
Tertile 3	49/703 (7.0)	0.67 (0.44–0.99)	0.75 (0.50–1.15)	0.74 (0.49–1.14)
*P* for trend	0.35	0.046	0.18	0.17
Each 1-μIU/ml increase in TSH	165/2103 (7.9)	0.97 (0.88–1.08)	1.00 (0.92–1.08)	1.00 (0.92–1.08)

Model 1: adjusted for age, sex, BMI at baseline

Model 2: further adjusted for triglyceride, HDL-c, diabetes and hypertension status, current smoking and drinking status, use of antihypertensive drugs, use of antidiabetic drugs, eGFR at baseline

Model 3: Further adjusted for TPOAb, TGAb at baseline

*P* for trend values for percentages of rapid eGFR decline are calculated by using Cochran–Mantel–Haenszel (CMH) method

Data are odds ratios (95% confidence intervals)

*BMI* body mass index, *eGFR* estimated glomerular filtration rate, *FT3* free triiodothyronine, *FT4* free thyroxine, *HDL-c* high-density lipoprotein cholesterol, *TSH* thyroid-stimulating hormone, *TPOAb* thyroid peroxidase antibody, *TGAb* thyroglobulin antibody

### Association of baseline serum FT4 levels with the predicted risks for future outcomes of CKD

According to the recommendations in the 2012 KDIGO clinical practice guideline for the evaluation and management of CKD [1], among the 198 participants who developed CKD during follow-up, 177 participants were at moderately increased risk of concurrent complications and further outcomes of CKD, 17 and 4 were at high risk and very high risk, respectively.

Table 4 shows associations of baseline serum FT4 levels with the predicted risks for future outcomes of CKD, among incident CKD individuals. After adjusting for a wide range of covariates, each 1-pmol/l increase and each 1-tertile increase of FT4 associated 11% and 30% increased risk for moderately increased risk of concurrent complications and further outcomes of CKD, respectively; and 22% and 161% increased risk for the combined high and very high increased risk of concurrent complications and further outcomes among incident CKD individuals, respectively.

**Table 4:** Association of baseline FT4 with predicted risks for future outcomes of incident CKD

	Predicted risks for future outcomes of incident CKD	
	Moderately increased risk	Combined high and very high increased risk
Case/number (%)	177/2103 (8.4)	21/2103 (1.0)
Each 1-pmol/l increase in FT4		
Model 1	1.12 (1.04–1.21)	1.18 (1.05–1.34)
Model 2	1.11 (1.03–1.19)	1.22 (1.06–1.41)
Each 1-tertile increase in FT4		
Model 1	1.33 (1.09–1.61)	2.73 (1.41–5.27)
Model 2	1.30 (1.06–1.59)	2.61 (1.32–5.16)

Model 1: adjusted for age, sex, BMI at baseline

Model 2: further adjusted for triglyceride, HDL-c, diabetes and hypertension status, current smoking and drinking status, use of antihypertensive drugs, use of antidiabetic drugs, urinary ACR, eGFR, TPOAb and TGAb at baseline

Data are odds ratios (95% confidence intervals), calculated by using multinomial logit regression analysis, referenced to low risk of prognosis of incident CKD

*ACR* albumin-to-creatinine ratio, *BMI* body mass index, *CKD* chronic kidney disease, *eGFR* estimated glomerular filtration rate, *FT4* free thyroxine, *HDL-c* high-density lipoprotein cholesterol, *TPOAb* thyroid peroxidase antibody, *TGAb* thyroglobulin antibody

## Discussion

In this community-based prospective investigation, we found that high levels of serum FT4 significantly associated with renal function decline in middle-aged and elderly Chinese. Major findings in our study were: (1) elevated levels of serum FT4 at baseline associated with increased risk of incident CKD and rapid eGFR decline; (2) serum FT4 positively associated with the increased risk for concurrent complications and further outcomes of CKD; (3) no significant associations of serum FT3, TSH with incident CKD or rapid eGFR decline were found in the present analysis.

In our study, we reported significant associations of FT4 with both incident CKD and rapid eGFR decline in a large general Chinese population, suggesting that when a higher FT4 level was found in clinical practice, a screening for kidney disease is warranted beyond hyperthyroidism treatments and the thyroid function monitoring. Our results were consistent with several previous findings. A prospective study focusing on thyroid function status and renal function in older persons found that overt hypothyroidism provided a protective effect on changes in renal function over time, whereas hyperthyroidism conveyed a harmful effect [21]. In addition, other perspective studies also demonstrated that overt and subclinical hypothyroidism in older age (>85 years old) might even have a protective effect, particularly on span of life [28, 29]. However, our findings were conflicted with the results from the Kangbuk Samsung Health Study (KSHS), which was performed among 104,633 Korean participants with an average age of 38.0 years old and normal thyroid function at baseline. It reported that high-normal levels of TSH and low-normal FT3 levels below 3 pg/ml, but not FT4 levels, were associated with increased risk of incident CKD, defined as eGFR <60 mL/min/1.73 m^2^ [20]. The disparate definitions of CKD might be a possible attributor for this discrepancy. In our study, we defined CKD not only on basis of eGFR but also on the presence of albuminuria. Albuminuria would be induced by high thyroid function and then could deteriorate renal function gradually [17, 30]. In addition, it might be due to the relatively older participants in our study than those in KSHS. With the increasing age, renal function declined [31] and thyroid function changed including increase of serum TSH and FT4, and decrease of total triiodothyronine [32].

In our study, we found that high levels of FT4, but not low levels of TSH, associated with incident CKD and rapid eGFR decline. Analogously, Ahmed MM reported that high levels of FT4, but not low levels of TSH, significantly associated with increasing severity of renal failure in patients with chronic renal failure and averagely aged 61 years old [33]. It might be partially due to the reduced sensitivity of pituitary with the increasing age; and then high FT4 levels could not generate negative feedback to TSH and suppress secretion of TSH [32, 34]. As shown in our study, serum FT4 was positively associated with age (*β* = 0.012, *P* = 0.0086), but TSH was not (*β* = −0.0021, *P* = 0.27).

However, the mechanisms of the positive associations of serum FT4 levels with incident CKD and rapid eGFR decline remain unclear. It might be due to the fact that high thyroid hormone levels increased the permeability of the glomerular barrier, and subsequently led to proteinuria [35]. Several studies have reported that overt proteinuria was observed in hyperthyroid rats, as well as in Graves’ disease patients [36]. It may also be possible that the presence of albuminuria could promote renal function deterioration. Previous studies found that the elevated ACR, even within normal range, was associated with a faster decline in eGFR [37, 38].

The major strength of our study is the prospective nature. We also carefully performed adjustments for the potential confounders, including the autoimmune thyroid antibodies. Besides, the CKD was defined based on either eGFR <60 ml/min/1.73 m^2^ or presence of albuminuria recommended by the 2012 KDIGO clinical practice guideline. The rationales arise from the evidence that decrease eGFR or albuminuria were consistently associated with increased risk of death, end stage renal disease or cardiovascular mortality [39]. However, several limitations should be acknowledged. Firstly, the GFR was estimated by the 2009 CKD-EPI equation, rather than the gold standard of GFR measurement, a technetium 99m diethylene-triaminepentaacetic acid (^99m^Tc-DTPA) renal dynamic imaging method. However, the accuracy of CKD-EPI equation to estimate GFR has already been confirmed and validated in many previous studies [40, 41]. Secondly, urinary albumin and creatinine were measured using a single spot urine sample. However, it was more practicable in large epidemiological studies [6], and was highly agree with the 24-h urinary albumin excretion [42, 43]. Thirdly, there were only two measurements of eGFR rather than repeated measurements of eGFR during follow-up. Fourthly, the current study was conducted in middle-aged and elderly Chinese adults. Hence, more cautions are needed to generalize the findings to other age and ethnic groups.

## Conclusions

In conclusion, the present study indicated that a high level of serum FT4, rather than serum TSH and FT3, was associated with increased risk of incident CKD, rapid eGFR decline and increased risks of concurrent complications and further outcomes of CKD. Further studies aimed to validate our findings in other age and other ethnic groups, to examine the association of developing hyperthyroidism with incident CKD, and to explore related mechanisms are warranted in the future.

## Abbreviations

ACR: albumin-to-creatinine ratio
BMI: body mass index
CV: coefficients of variation
CKD: chronic kidney disease
eGFR: estimated glomerular filtration rate
FPG: fasting plasma glucose
FT3: free triiodothyronine
FT4: free thyroxine
HDL-c: high-density lipoprotein cholesterol
LDL-c: low-density lipoprotein cholesterol
OGTT: oral glucose tolerance test
OR: odds ratio
SD: standard deviation
TC: total cholesterol
TSH: thyroid-stimulating hormone
TPOAb: thyroid peroxidase antibody
TGAb: thyroglobulin antibody
